# Optimal injection rate of ultrasound contrast agent for evaluation of focal liver lesions using an automatic power injector: a pilot study

**DOI:** 10.1186/s40064-016-2364-5

**Published:** 2016-06-17

**Authors:** Katsutoshi Sugimoto, Fuminori Moriyasu, Hirohito Takeuchi, Mayumi Kojima, Saori Ogawa, Takatomo Sano, Yoshihiro Furuichi, Yoshiyuki Kobayashi, Ikuo Nakamura

**Affiliations:** Department of Gastroenterology and Hepatology, Tokyo Medical University, 6-7-1 Nishishinjuku, Shinjuku-ku, Tokyo 160-0023 Japan

**Keywords:** Ultrasound, Contrast, Injection rate, Focal liver lesion, Power injector

## Abstract

**Objective:**

To determine the optimal bolus injection rate of ultrasound (US) contrast agent in vascular imaging for focal liver lesions.

**Methods:**

Thirteen patients with 13 focal liver lesions (5 hepatocellular carcinomas (HCCs) with cirrhosis, 4 liver metastases, 2 hemangiomas, 1 intrahepatic cholangiocarcinoma, 1 focal nodular hyperplasia) received two bolus injections of Sonazoid (at 0.5 and 2.0 mL/s) using an automatic power injector. The lesion-to-liver contrast ratio at peak enhancement was quantitatively evaluated. Enhancement of the lesions compared to liver parenchyma was assessed by two independent readers using a five-point scale and qualitatively evaluated by receiver operating characteristic (ROC) analysis.

**Results:**

For all lesions, the contrast ratio was not significantly different between the two injection rates. For HCCs, the contrast ratio was higher at 0.5 mL/s (7.41 ± 6.56) than at 2.0 mL/s (4.28 ± 4.66, *p* = 0.025). For all lesions, the mean area under the ROC curve (AUC) was not significantly different between the two injection rates. For HCCs, the AUC was greater at 0.5 mL/s than at 2.0 mL/s (AUC: 0.86, *p* = 0.013).

**Conclusion:**

In contrast-enhanced US, an injection rate of 0.5 mL/s is superior to an injection rate of 2.0 mL/s for the quantitative and qualitative analysis of HCCs in the cirrhotic liver.

## Background

Current low-mechanical index (low-MI) techniques for contrast-enhanced ultrasound (CEUS) using second-generation microbubble agents have advantages in the characterization of focal liver lesions (FLLs), including the real-time observation of continuous hemodynamic changes in hepatic lesions (Claudon et al. 2013). However, to our knowledge, there is little agreement concerning the appropriate injection rate for microbubble agents in CEUS, despite the fact that there have been many studies regarding the injection rate of contrast media in dynamic computed tomography (CT) (Tublin et al. 1999) and magnetic resonance (MR) imaging (Schmid-Tannwald et al. 2012).

We have therefore conducted a prospective pilot study to determine both qualitatively and quantitatively the optimal bolus injection rate of US contrast agent in vascular imaging for the evaluation of FLLs, with the ultimate goal of establishing suitable protocols for the examination of patients with FLLs.

## Methods

### Subjects

Thirteen consecutive patients (2 men and 11 women; age, 67.5 ± 10.4 years (mean ± standard deviation); body weight, 51.9 ± 9.4 kg; and height, 151.8 ± 8.0 cm) with FLLs to be evaluated by CEUS were enrolled as the subjects of this pilot study. Five subjects had hepatocellular carcinoma (HCC) with cirrhosis, 4 had liver metastasis (2 from pancreatic neuroendocrine tumor and 2 from breast cancer), 2 had hemangioma, 1 had cholangiocarcinoma, and 1 had focal nodular hyperplasia (FNH).

The diagnosis of HCC, as well as liver cirrhosis, all metastases, and cholangiocarcinoma, was based on the findings of needle biopsy (21-gauge Majima needle; Top Surgical Manufacturing, Tokyo, Japan). The diagnosis of hemangioma and FNH was based on a combination of imaging findings: a classic contrast enhancement pattern observed on CEUS, with peripheral nodular enhancement or tiny enhancing dots in the early phase and persistent enhancement or centripetal fill-in enhancement in the late and postvascular phases for hemangioma (Claudon et al. 2013), and with a hypervascular appearance with the presence of stellate lesional vessels with a centrifugal filling direction in the early phase and persistent enhancement in the late and postvascular phases for FNH (Claudon et al. 2013).

### CEUS imaging

A diagnostic US system (TSU-A500, Aplio™ 500; Toshiba Medical System Corporation, Tochigi, Japan) with a 3.75-MHz convex transducer (PVT-375BT; Toshiba) was used in this study. The imaging mode was wideband harmonic imaging (commercial name “Pulse subtraction”) with transmission and reception frequencies of 1.75 and 3.5 MHz, respectively.

All subjects were fasted overnight. All US scans were performed by the same sonologist (M.A.) with 5 years of experience in standard US and CEUS. The probe was held steady during acquisition while the subject was breathing shallowly and evenly in order to obtain a single image showing the largest plane through the lesion and including adjacent liver parenchyma.

**Fig. 1:**
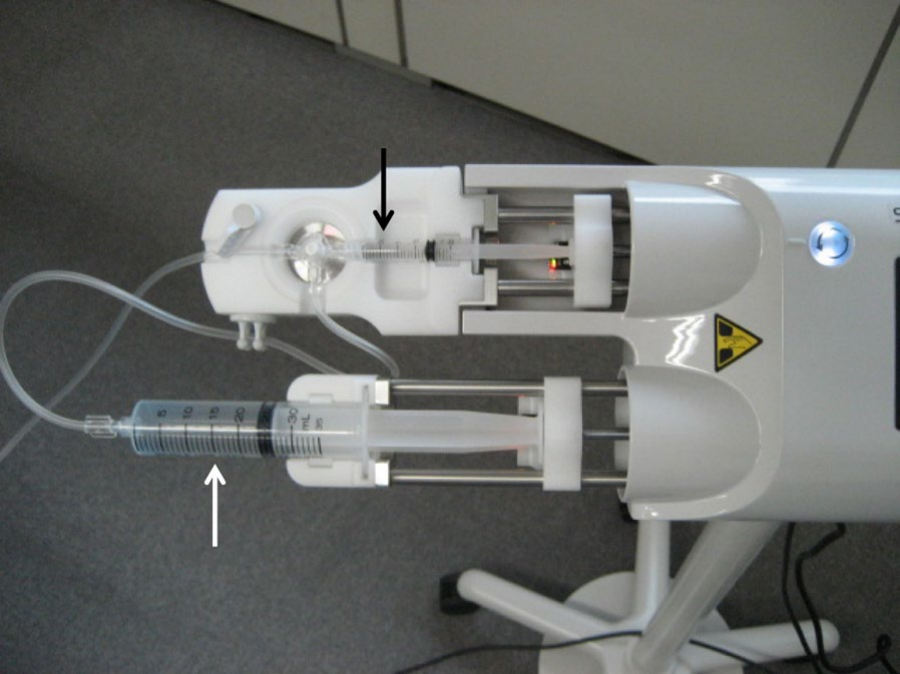
The automatic power injector employed in the present study. It consists of a double syringe pump capable of injecting microbubble contrast agent (*white arrow*) and saline (*black arrow*) sequentially

A second-generation US contrast agent (Sonazoid; Daiichi-Sankyo, GE, Tokyo, Japan) was used. Each subject received two injections on the same day. The first bolus injection was 0.5 mL of Sonazoid injected at 0.5 mL/s followed by a 10-mL saline flush injected at the same rate, and the second bolus injection was 0.5 mL of Sonazoid injected at 2.0 mL/s followed by a 10-mL saline flush injected at the same rate. All subjects received injection into an antecubital vein via a 21-gauge intravenous catheter using an automatic power injector (SONAZOID SHOT™; Nemoto Kyorindo, Tokyo, Japan) consisting of a double syringe pump capable of injecting microbubble contrast agent and saline sequentially (Fig. 1). It should be noted that before the second injection, the remaining circulating microbubbles were destroyed as completely as possible by performing high-MI US scanning for at least 20 min. The automatic injector system used in the present study supports variable injection speeds with manual or pedal control. The microbubble syringe was automatically shaken before microbubble injection when the system was stopped in order to ensure effective injection. The two syringes were connected to a T-line, and the injection speed was adjustable in the range of 0.1–2.0 mL/s.

Examination recording and timing were triggered immediately after the contrast agent was injected. The acquisition time for the raw data was 1 min. The use of low-MI nonlinear imaging (MI < 0.2) allowed the microbubbles to be visualized nondestructively with simultaneous low-MI B-mode anatomic imaging displayed side by side.

CEUS examinations were performed at a rate of 10 frames per second and with a dynamic range of 60 dB. The receive gain, image depth, and transmit focus were optimized for each subject. When the examination was completed, the raw data were stored in a workstation (UltraExtend FX; Toshiba). Initial quantification was performed using US quantification software (CHI-Q; Toshiba).

### Region of interest (ROI) analysis

**Fig. 2:**
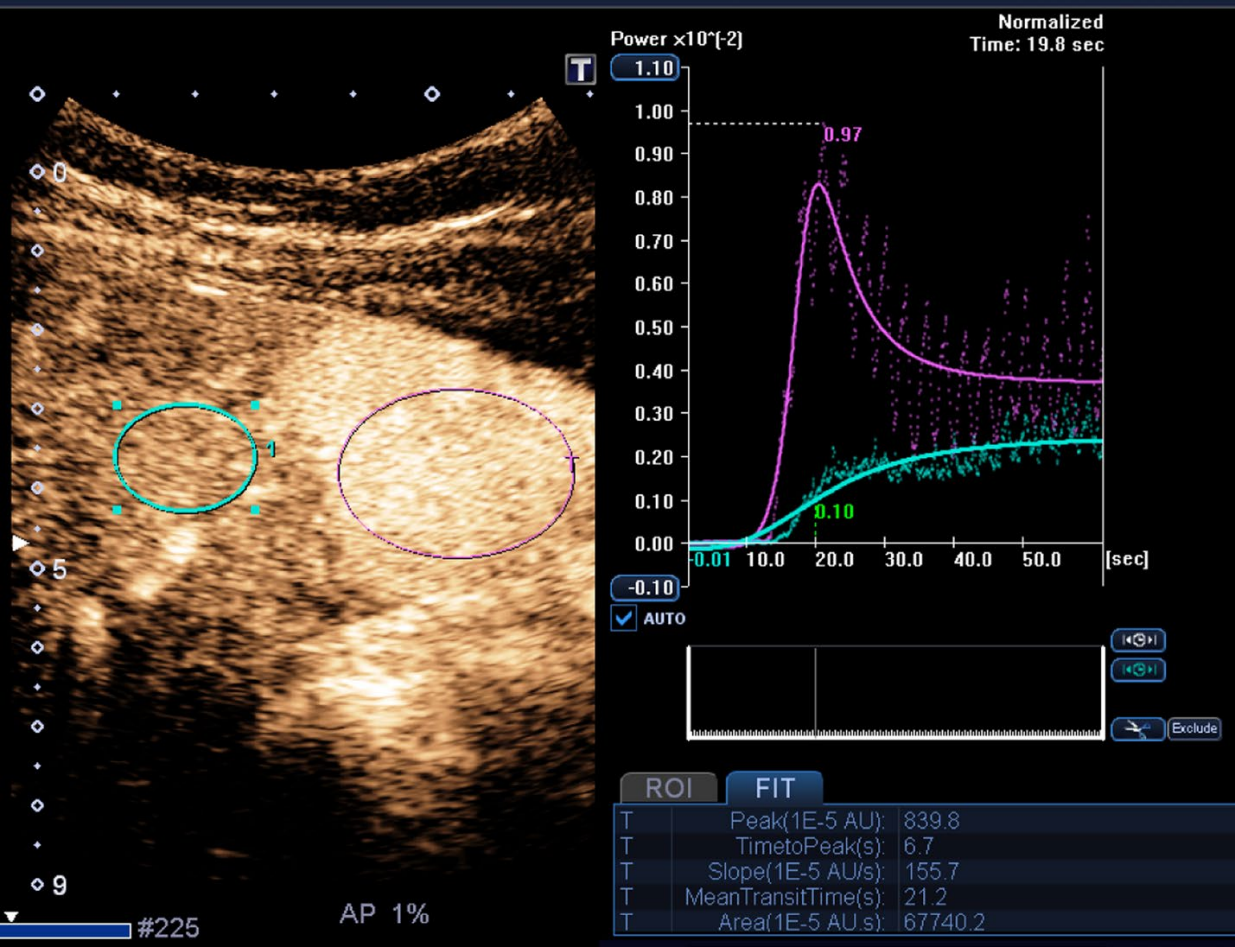
Example of the workstation interface. A *red* circular region of interest is placed on the lesion (focal nodular hyperplasia) and a *blue* region of interest is placed in adjacent liver parenchyma. Time-intensity curves of the lesion (*red* *curve*) after modelization and of adjacent liver parenchyma (*blue* *curve*) after modelization are displayed on the *right side*

After the raw data were transferred to the workstation, the data were analyzed and ROIs encompassing the lesions were drawn by the same hepatologist (K.S.). Similarly, a circular or oval ROI was placed in adjacent liver parenchyma at the same depth as the lesion. This ROI was as large as possible and placed so as to avoid vessels and artifacts. An example showing how the ROIs were placed is shown in Fig. 2. The time-intensity curve of the total surface delimited was calculated as the sum of the time-intensity curves of all pixels using the linear raw data obtained with the CHI-Q software.

### Quantitative perfusion parameter analysis

The fitting of the curve was based on a mathematical model developed by the Gustave Roussy Institute (patent WO/2008/053268, “Method and System for Quantification of Tumoral Vascularization”), and the Solver program in Excel was used to determine the coefficients of the fitted curve based on the least squares method. This method consists of minimizing the differences between the parameters of the unfitted raw curve and the coefficients of the Gustave Roussy Institute equation (Gauthier et al. 2012):

$$I\left( t \right) = a_{0} + (a_{1} - a_{0} )* \left( {\frac{{A + \left( {\frac{t}{{a_{2} }}} \right)^{p} }}{{B + \left( {\frac{t}{{a_{2} }}} \right)^{q} }} } \right)$$

where *I*(*t*) describes the variation in the intensity of contrast uptake as a function of time, *a*_0_ is the intensity before the arrival of the contrast agent, *a*_1_ is linked to the maximum value of contrast uptake, *a*_2_ is linked to the rise time to the peak intensity, *p* is a coefficient related to the increase in intensity, *q* is a coefficient related to the decrease in intensity, and *A* and *B* are arbitrary parameters.

**Fig. 3:**
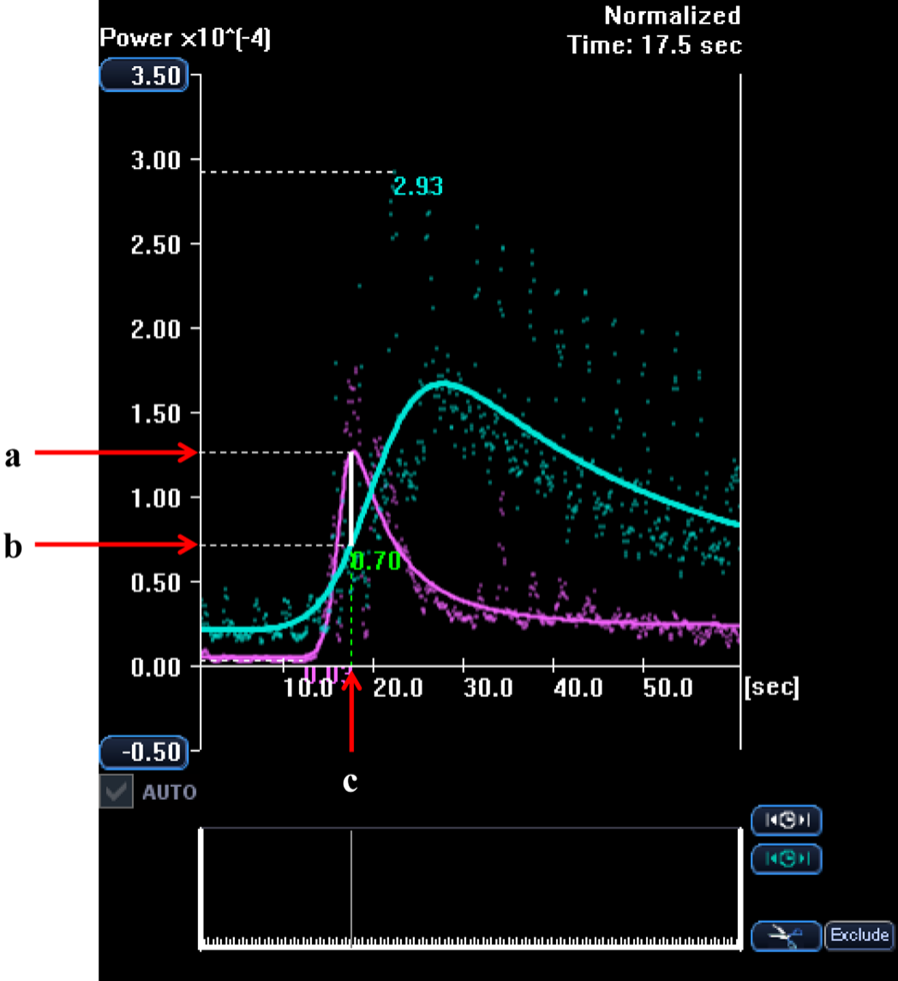
The lesion-to-liver contrast ratio is calculated using the following equation: lesion-to-liver contrast ratio equals “*a*” divided by “*b*”. *a* Peak intensity of the lesion (in arbitrary units), *b* intensity of the liver parenchyma at the time to peak intensity of the lesion, *c* time to peak intensity of the lesion (in seconds)

In this study, two semiquantitative perfusion parameters were extracted from the time-intensity curves after modelization: the peak intensity of the FLL (in arbitrary units) and the time to peak intensity (in seconds). The lesion-to-liver contrast ratio was calculated using the following equation: the lesion-to-liver contrast ratio equals the peak intensity of the lesion divided by the intensity of the liver parenchyma at the time to peak intensity of the lesion. The definitions are shown in Fig. 3. All quantifications were performed by the same author (K.S.).

### Qualitative visual analysis

Two experienced sonologists (M.A. and S.O.) involved in the study qualitatively assessed the degree of lesion conspicuity as compared to adjacent liver parenchyma in CEUS images during all vascular phases. The two sonologists performed assessment independently and were blinded to the injection rate used.

All image cine clips were reviewed at the workstation (UltraExtend FX; Toshiba). The degree of lesion conspicuity was visually scored in comparison to adjacent liver parenchyma using a five-point scale: 5 (excellent), contrast enhancement provided optimal information for establishing a diagnosis; 4 (good), contrast enhancement provided adequate information for establishing a diagnosis; 3 (fair), contrast enhancement provided acceptable information for establishing a diagnosis, but image quality was unsatisfactory; 2 (poor), contrast enhancement did not provide adequate information for establishing a diagnosis; and 1 (none), no enhancement was observed.

### Statistical analysis

The results were expressed as mean ± standard deviation. The quantitative perfusion parameter analysis results for the two injection rates were compared using the two-tailed Student’s *t* test. The conspicuity of each lesion at each injection rate and for each reviewer was evaluated by receiver operating characteristic (ROC) analysis. The area under the ROC curve (AUC) was calculated for each reviewer and for each injection rate. The two-tailed Student’s *t* test was also used to test for differences in the AUCs. A *p* value of less than 0.05 was considered to be statistically significant. All statistical analyses were performed using a computer software package (JMP^®^ version 11; SAS Institute Japan Ltd., Tokyo, Japan).

## Results

### Quantitative assessment

**Fig. 4:**
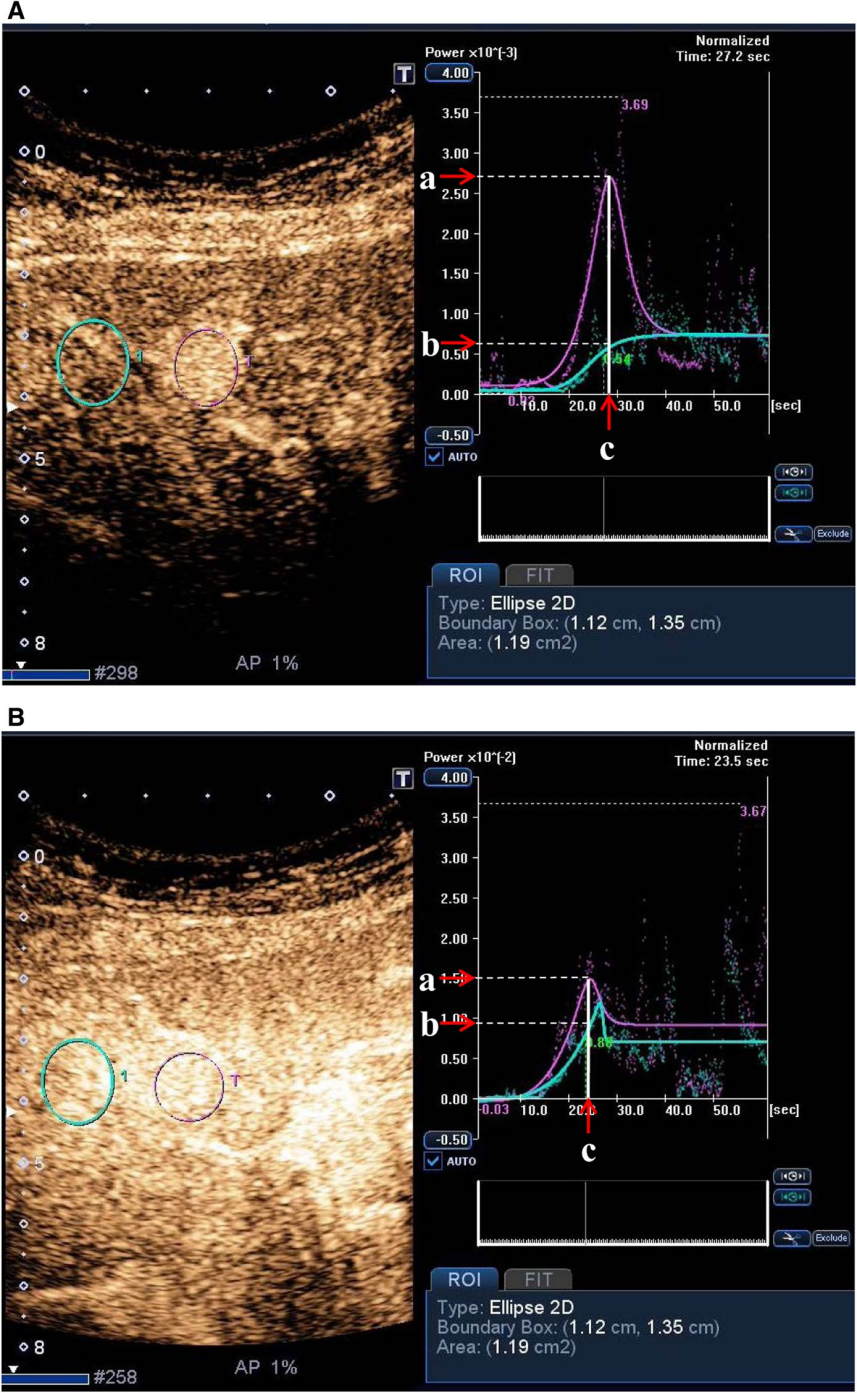
Images of an 81-year-old woman with liver cirrhosis and a hepatocellular carcinoma (HCC) in segment 4. **A** Sonazoid is injected at a rate of 0.5 mL/s. The CEUS image at peak enhancement shows clear hypervascularity; the resulting average visual rating is 4.5 (reader 1: 4, reader 2: 5). A *red* circular region of interest is placed on the HCC and a *blue* region of interest is placed in adjacent liver parenchyma. The lesion-to-liver contrast ratio is 4.70. **B** Sonazoid is injected at a rate of 2.0 mL/s. The CEUS image at peak enhancement shows isovascularity to slight hypervascularity; the resulting average visual rating is 3 (reader 1: 3, reader 2: 3). A *red* circular region of interest is placed on the HCC and a *blue* region of interest is placed in adjacent liver parenchyma. The lesion-to-liver contrast ratio is 1.80

For all lesions, no significant difference in the lesion-to-liver contrast ratio was observed between the two injection rates. For HCC, the contrast ratio was higher for injection at 0.5 mL/s (7.41 ± 6.56) than for injection at 2.0 mL/s (4.28 ± 4.66, *p* = 0.025) (Fig. 4). The time to peak intensity was significantly shorter for injection at 2.0 mL/s (21.6 ± 6.0 s) than for injection at 0.5 mL/s (27.1 ± 4.1 s, *p* = 0.0057).

### ROC analysis

For all lesions, no significant difference in the mean AUC was observed between the two injection rates. For HCC, the mean AUC was greater for injection at 0.5 mL/s than for injection at 2.0 mL/s (AUC: 0.86, *p* = 0.013) (Fig. 4).

## Discussion

An important advantage of CEUS is the ability to assess contrast enhancement patterns in real time with a much higher temporal resolution than is possible with other imaging modalities. It may therefore seem that there is no need to define an optimal injection protocol, unlike the case for CT or MR imaging.

In fact, one set of clinical CEUS guidelines for the liver simply states that ultrasound contrast agent should be administered as a bolus injection followed by a 0.9 % normal saline flush (Claudon et al. 2013). Another states that contrast agent should be administered by bolus injection within 2 s, followed by flushing with 10 mL of normal saline (Jang et al. 2013). We basically agree with these guidelines, but we also feel that it is important to conduct studies to determine whether it is possible to develop optimal CEUS examination protocols, particularly with regard to the injection of contrast agent.

The results of this pilot study showed that, for all lesions, no significant differences were observed between the two injection rates in both quantitative and qualitative analysis. However, for HCC in the cirrhotic liver, an injection rate of 0.5 mL/s was found to be significantly superior to an injection rate of 2.0 mL/s in both quantitative and qualitative analysis.

Elucidating the mechanisms responsible for the finding that a slow injection rate is superior only for HCC in patients with a cirrhotic liver may be important in CEUS examinations for the characterization of FLLs. In general, it is said that as the liver becomes cirrhotic, the hepatic blood flow balance changes from “portal vein dominant” to “hepatic artery dominant” (Kleber et al. 1999). Thus, we assume that the arterial enhancement of HCC can be masked by that of adjacent liver parenchyma and that this phenomenon is expressed more strongly at a faster injection rate because the time to peak intensity is significantly shorter for a fast injection rate than a slow injection rate. Our visual assessment and ROC analysis results support this theory.

Our study has several limitations. First, the number of subjects was relatively small, although the number is probably sufficient for a pilot study. Second, pathologic confirmation was not obtained for a minority of the lesions, although all subjects did undergo confirmatory imaging examinations.

In conclusion, in CEUS examinations, an injection rate of 0.5 mL/s was found to be significantly superior to an injection rate of 2.0 mL/s for the quantitative and qualitative analysis of HCC in the cirrhotic liver. The use of a slow injection rate may improve the diagnostic accuracy of CEUS for HCC in patients with cirrhosis of the liver.
